# The impact of fires on mosquito populations in Eastern Siberian forests

**DOI:** 10.1371/journal.pone.0326366

**Published:** 2025-09-08

**Authors:** Alexey V. Shvetsov

**Affiliations:** 1 Togliatti State University, Togliatti, Russia; 2 Moscow Polytechnic University, Moscow, Russia; 3 North-Eastern Federal University, Yakutsk, Russia; 4 RUDN University, Moscow, Russia; National Cheng Kung University, TAIWAN

## Abstract

Forest fires have a significant impact on forest fauna, killing not only mammals and birds, but also less noticeable representatives of forest fauna – insects. Existing research have mainly studied the effects on vertebrate taxa, but the data on the effects of fires on the number of insects living in forests is currently insufficient to cover all the groups. The research presented in this paper examines the immediate impact of forest fires on the number of adults in mosquito populations (*Culicidae*) in burned areas of the boreal forest. The data were collected in the boreal forests of Eastern Siberia, in the Republic of Sakha (Yakutia). Field survey was conducted in ten forest areas, including five recently burned and five unburned areas. Mosquitoes were caught in flight and counted during daylight hours within the time span of 7 days after the fire. We compared the data obtained from burned and unburned adjacent forest plots, which allowed us to establish the level of immediate impact of forest fires on the abundance of adult mosquito populations in the burned plots of the boreal forest. In all five pairs of forest plots, a decrease in the abundance of adult mosquitoes was noted, within the range of 86.6–93.9%. The data clearly show a lower number of adult mosquitoes for several days after the forest fire. The results obtained are of interest for further studying, regardless of the medical significance of mosquitoes, with consideration of their role in boreal ecosystems. This study is planned to be continued over the next few years in order to obtain new data on the impact of forest fires on mosquito populations in a medium and long term, which will allow us to form a broader vision of this problem.

## 1. Introduction

Boreal forests extend in two transcontinental belts across North America and Eurasia covering an area of 12 million square kilometers in Scandinavia and Russia (two-thirds), Canada and Alaska (one-third) mainly between 70° and 45° north latitude [1]. These forests are mainly dominated by coniferous trees such as pine (*Pinus*), spruce (*Picea*), fir (*Abies*), larch (*Larix*), mixed with more sparse deciduous trees such as birch (*Betula*), poplar (*Populus*), etc. [1]. Forests of this kind are particularly liable to destruction by fire [2] as the ground under the trees in summer months is covered with dry grass and dry lichen Cladonia rangiferina. Such lichen is very susceptible to ignition, which contributes to the rapid spread of fire. The resin emitted by coniferous trees is also a highly flammable material, that releases a significant amount of heat energy in the process of combustion. These factors create suitable conditions for the emergence of forest fires in Eastern Siberia.

Forest fires, in which sometimes the territory within a radius of tens of kilometres is burned out, have a significant negative impact on forest fauna [3,4]. This impact is explained by the fact that the average maximum temperature during a forest fire on the surface of mineral soil reaches 128°C, while the fire spread speed can reach up to 250 m/min [5].

Forest fires kill many animals, but available studies have mostly examined the effects on vertebrate taxa [6,7], but the data on the effects of fires on forest-dwelling insects is currently insufficient to cover all the groups. Among the works on this topic are [8,9], which examine the effects of fire on insects such as the pyrophilous beetle *Monochamus* (*Cerambycidae*) and the Australian fire-beetle *Merimna Atrata* that benefit from forest fires, and [10] which examines the taxonomic and functional response of ant species to fire in forest landscapes.

Mosquitoes are among the most widespread insects in forests and are an important part of the food chain in nature [11,12] and are part of the biomass that feeds fish, birds and amphibians, which in turn feed larger forest mammals. In this context, it is important to study the impact of fire on mosquito abundance. Forest fires may kill adult mosquitoes both flying and sitting on vegetation. In addition to adult mosquitoes, fires can affect the number of mosquito larvae in puddles and water bodies. This is possible due to contamination of water with combustion products such as soot and ash [13,14], containing toxic organic compounds PAHs [15,16], in potentially dangerous concentrations.

The objective of this study is to investigate the immediate effects of forest fires on adult abundance in mosquito populations in burned areas of boreal forest.

To address this objective, a field survey was conducted in which data were collected from ten forest plots, which included five recently burned and five unburned plots. Data were collected at a time interval of 7 days post-fire. It is expected that comparison of data from burned and unburned bordering forest plots will indicate the level of immediate effect of forest fires on mosquito adult abundance.

## 2. Materials and methods

This study was conducted in a burned boreal forest in Eastern Siberia (Republic of Sakha, Yakutia, North-Eastern Russia), which is one of the largest administrative-territorial regions of the planet with an area of 3,103,200 km^2^. The region is almost completely covered with forests [17] and has a high fire activity [18]. Thus, the largest known forest fire in the history of the planet occurred on the territory of Yakutia in 2003 [19], when 22 million hectares of forest burned.

### 2.1. Mosquito communities

Fifteen species of mosquitoes of the family *Culicidae* are distributed throughout the forests of Eastern Siberia, belonging to the genera *Culiseta, Aeded* and *Anopheles* [20].

Mosquitoes are present in the forests of Eastern Siberia during the warm part of the year, from May to September.

### 2.2 Territory

**Fig 1:**
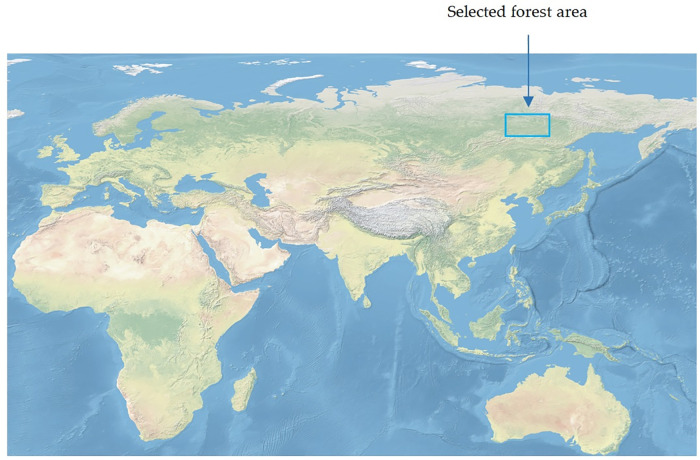
The forest area selected for the study (map: https://www.naturalearthdata.com).

The field survey was conducted in a forested area located in the quadrant labelled in Fig 1.

Data collection during the field survey was carried out in ten forest plots, including five recently burned and five unburned plots. Primary data from the plots are presented in the Supporting Information file [21].

All plots were grouped into five pairs (two plots per pair) in which the burned and unburned plots were adjacent, and all plot pairs were located in the same forest area. The distances between neighboring plot pairs varied from 2 to 10 km. Data from each plot pair were collected within a time span of 7 days after the fire in the burned area.

The total area of burned plots is ~ 3000 hectares. The forest type on the plots is boreal forest. The species composition of the plots is based on larch (~ 80%), mainly Gmelina Larch (*Lárix gmélinii*) and Siberian Larch (*Lárix sibírica*) with the addition of trees such as Siberian Spruce (*Pícea obováta*), Siberian Pine (*Pínus sylvéstris*), Siberian Cedar (*Pínus sibírica*) (~ 7%), and Stone Birch (*Betula ermanii*) (~ 1%). The plots are mainly dominated by mature and overmature (100 years and older) trees (~ 40% of the area). Mean annual temperatures ranges from −42°C to 25°C and rarely fall below −49°C or above 31°C. Temperature during sample collection was from 15°C to 24°C. Average annual precipitation fluctuates within the range of 150–200 mm. Elevation is 347-417m.

### 2.3 Data collection

Sampling was done by capturing adult mosquitoes in flight, a special entomological net was used for capturing by placing the captured mosquitoes in removable bags. The index of mosquito abundance at capture was calculated using the following method: 10 swings of the net (figure-eight movement) followed by repeat in five minutes (using a new bag) [22]; each capture resulted in two samples.

Mosquitoes were captured during daylight hours, five times a day, 1–2 hours apart, during the period when mosquitoes are most active from 09.00 to 16.00. As a result, 10 samples were taken at each forest plot during one daylight period, including data on the number of captured adult mosquitoes. When counting the number of mosquitoes, adult mosquitoes of the family *Culicidae*, belonging to the following genera: *Culiseta, Aedes* and *Anopheles*, were included in the total number of captured insects.

**Fig 2:**
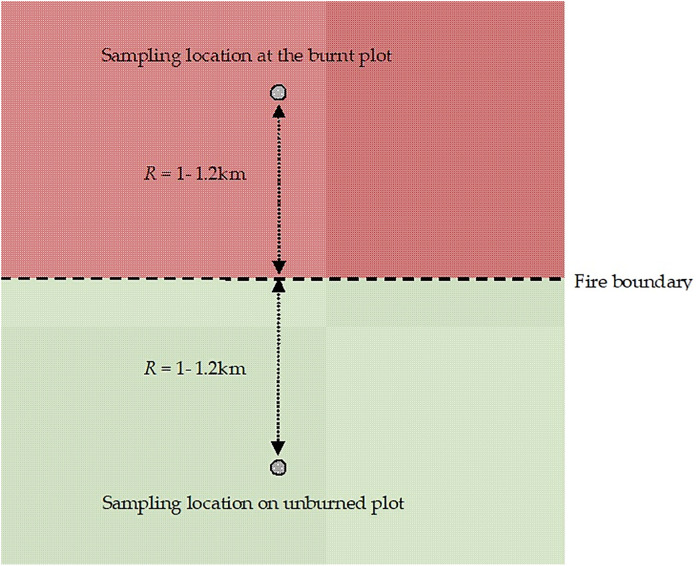
Scheme of sampling from two plots belonging to the same pair.

Sampling was conducted in June and July 2024, with sampling in a separate pair of plots conducted during the same daylight hours, at both bordering plots (burned and unburned) simultaneously, and following the pattern shown in Fig 2.

The distance from the measurement site to the boundaries of the burned area (Fig 2), low winds, and the absence of animal odours that lure mosquitoes from the burned areas suggests that the probability of mosquitoes arriving in the fire area from neighbouring forests is low.

The conditions during data collection were as follows: during data collection, there was no precipitation in the burned and unburned areas, cloud cover was 3–7, wind was up to 3 m/s, fire type observed in the burned areas was continuous, fire intensity was high, there were ~ 70% standing charred tree trunks in the areas, and safe sampling was possible.

## 3. Results

The total number of mosquitoes captured in unburned plots was 273 units; the total number of mosquitoes captured in burned plots was 25 units.

**Fig 3:**
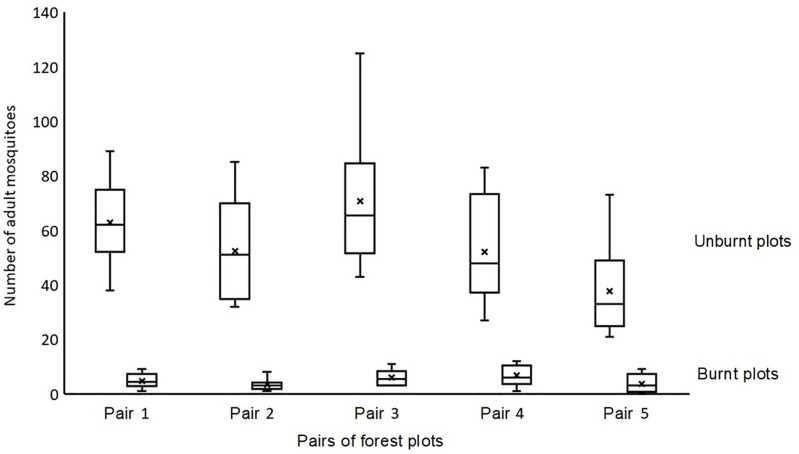
Data obtained from the five pairs of forest plots.

The average and standard deviation of mosquitoes captured per sampling unit, are shown in Fig 3.

We took the median value for mosquito abundance in each pair of forest plots to perform analyses of the observations conducted (Table 1).

**Table 1:** Median data on the number of mosquito adults collected.

Pair of forest plots	Median value of mosquito abundance in samples, unburned forest plot, units	Median value of mosquito abundance in samples, in the burned forest plot, units
The first	63	5
The second	49	3
The third	71	6
The fourth	52	7
The fifth	38	4

The ANOVA results (based on data from Table 1) *F*_*emp*_ = 9.132.


$$F_{cr}=5.32<F_{emp}$$

The differences in mosquito abundance in the absence and after fire are greater than random differences within each group (α = 0.05). Thus, the consequences of fire affect the number of mosquitoes.

## 4. Discussion

The data obtained confirm that forest fires have an immediate effect on the number of adults in mosquito populations in burned areas of boreal forest. All five pairs of forest plots showed a decrease in mosquito adult abundance, ranging from 86.6–93.9%. The data clearly show lower numbers of adult mosquitoes in the days following a forest fire. However, it is difficult to draw conclusions about mosquito mortality rates or their impact on the forest ecosystem. This is an interesting point to consider, regardless of the medical importance of mosquitoes, given their role in boreal ecosystems. Analyzing the data collected, it can be concluded that the amount of biomass serving as food for fish, birds and amphibians decreases in the short term in burned forest areas. Extrapolation of the decrease in the number of adult mosquitoes in flight relative to the total number of adult mosquitoes in the populations in the burned forest areas is due to the fact that trapping and counting of adult mosquitoes was carried out during daylight hours, when mosquitoes have the highest flight activity, i.e., the majority of adult mosquitoes are in flight.

By analyzing the difference in mosquito numbers between burned and unburned areas, it can be said that the decrease in numbers in burned areas may be due to both mosquito mortality from fire and movement of mosquitoes from burned to unburned areas. Adult mosquitoes can fly > 2 km (strong flyers) and <2 km weak flyers [23], which theoretically allows them to cover the distance between measurement points in burned and unburned areas (Fig. 2).

The necessity to take into account the data presented in this paper is determined by the fact that as a result of a decrease in mosquito numbers, a “domino effect” may occur, causing further changes in the forest fauna. The decrease in mosquito numbers may eventually lead to a decrease in mosquito predators in burned areas, such as the following species: *Plecotus ognevi, Eptesicus nilssonii, Myotis sibiricus, Salamandrella keyserlingii, Rana amurensis, Rana chensinensis, Rana arvalis* and others. On the other hand, these species may also eventually be killed by fire. Given that forest fires in Eastern Siberia occur annually and cover millions of hectares, the cumulative impact on the fauna may eventually be quite significant.

The reduction of mosquito population may have a positive value, as mosquitoes are one of the main factors in reducing the popularity of eco-tourism, they also have a depressing effect on farm animals, including such as Reindeer (lat. Rangifer tarandus), Yakut horses, etc. It should be taken into account that eco-tourism and farm animal breeding are one of the basic directions in the economy of Yakutia [24,25]. In this context, an interesting question for further research is how long the mosquito population decline can last after a fire, in other words, when mosquitoes can recolonize burned areas, depending on the duration of the mosquito life-cycle. Such data can be obtained by making repeated observations in previously surveyed burned forest plots. The ideal conditions for obtaining such data would be if samplings were carried out under similar climatic conditions, at similar periods of the year, over several years, with a frequency of once a year. In the context of analyzing the prospects of mosquitoes repopulating burned areas, it should also be noted that forest fires destroying the vegetation cover that protects the soil from the thermal effects of the sun eventually lead to thawing of permafrost. The thawing of permafrost, on which the forests of Eastern Siberia are located, leads to an increase in the number of water bodies and waterlogged soil, which in turn expands the breeding ground for mosquitoes [26,27]. Therefore, the effect of forest fires on mosquito populations may be longer lasting, and in the long term may lead to the opposite effect, which is to increase mosquito populations by increasing the number of water bodies suitable for their breeding.

## 5. Conclusions and future work

This paper presents data regarding the immediate effects of forest fires on adult abundance in mosquito populations in burned areas of boreal forests. The data are primarily aimed at researchers working in the field of wildlife conservation, but may also be useful to researchers and organizations involved in wildfire prevention. The data can be used in ecological and research programs aimed at analyzing the transformation of forest ecosystems as a result of fires.

The study presented in this paper is intended to be continued over the next few years to provide new data on the effects of forest fires on mosquito abundance in the medium to long term. An interesting aspect to expand the scope of the work initiated is the study of the effects of forest fires on other insect communities.
